# A phase I study comparing the biosimilarity of the pharmacokinetics and safety of recombinant humanized anti-vascular endothelial growth factor monoclonal antibody injection with Avastin^®^ in healthy Chinese male subjects

**DOI:** 10.1186/s40360-023-00673-y

**Published:** 2023-05-27

**Authors:** Hongtao Li, Xiangdi Zhao, Jing Xie, Xingyu Zhu, Yue Su, Cuixia He, Jiaxiang Ding, Minhui Zhu, Yuanyuan Xu, Ying Wang, Rongfang Shan, Bingyan Liu, Yuzhou Ding, Yuanyuan Liu, Huan Zhou, Yunqiu Xie

**Affiliations:** 1 National Institute of Clinical Drug Trials, The First Affiliated Hospital of Bengbu Medical College, Bengbu, Anhui China; 2 Department of Surgical Oncology, The First Affiliated Hospital of Bengbu Medical College, Bengbu, Anhui China; 3 School of Pharmacy, Bengbu Medical College, Bengbu, Anhui China; 4 School of Public Foundation, Bengbu Medical College, Bengbu, Anhui China; 5 Department of Critical Care Medicine, The First Affiliated Hospital of Bengbu Medical College, Bengbu, Anhui China

**Keywords:** Bevacizumab, Biosimilarity, Immunogenicity, Pharmacokinetics, Recombinant humanized anti-vascular endothelial growth factor monoclonal antibody injection

## Abstract

**Background:**

The biosimilar landscape for malignancies continues to grow, with several biosimilars for reference product bevacizumab currently available. Bevacizumab has been shown to be well tolerated; however, the safety of recombinant humanized anti-vascular endothelial growth factor (VEGF) monoclonal antibody injection remains unclear. This study aimed to compare the pharmacokinetics (PK), safety, and immunogenicity of recombinant humanized anti-VEGF monoclonal antibody injection to that of Avastin® in healthy Chinese male volunteers.

**Methods:**

A randomized, double-blind, single-dose, and parallel-group study was performed on 88 healthy men who randomly (1:1) received either the test drug as an intravenous infusion of 3 mg/kg or Avastin®. The primary PK parameter was area under the serum concentration-time curve (AUC) from time zero to last quantifiable concentration (AUC_0–t_). Secondary endpoints included maximum observed serum concentration (C_max_), AUC from 0 extrapolated to infinity (AUC_inf_), safety, and immunogenicity. Serum bevacizumab concentrations were measured using a validated enzyme-linked immunosorbent assay (ELISA).

**Results:**

The baseline characteristics were similar among the two groups. The 90% confidence interval (CI) for the geometric mean ratio of AUC_0–t_, C_max_ and AUC_inf_ between the test group and reference group were 91.71%–103.18%, 95.72%–107.49% and 91.03%–103.43%, respectively. These values were within the predefined bioequivalence margin of 80.00%–125.00%, demonstrating the biosimilarity of the test drug and Avastin®. Eighty-one treatment-emergent adverse events were reported, with a comparable incidence among the test group (90.91%) and the reference group (93.18%). No serious adverse events were reported. The incidence of ADA antibodies in the two groups was low and similar.

**Conclusion:**

In healthy Chinese men, PK similarity of recombinant humanized anti-VEGF monoclonal antibody injection to Avastin® was confirmed, with comparable safety and immunogenicity. Subsequent studies should investigate recombinant humanized anti-VEGF monoclonal antibody injection in patients setting.

**Trial registration:**

Registered 08/10/2019, CTR20191923.

**Supplementary Information:**

The online version contains supplementary material available at 10.1186/s40360-023-00673-y.

## Background

Bevacizumab (Avastin®), produced in Chinese hamster ovary cells using DNA technology, is a recombinant humanized immunoglobulin G1 monoclonal antibody. Bevacizumab works by preventing vascular endothelial growth factor (VEGF) from binding to its receptors (Flt-1 and KDR) on the surface of endothelial cells, preventing angiogenesis and tumor growth [1]. Furthermore, bevacizumab inhibits VEGF activity, resulting in the normalization of abnormal tumor tissue structure, microenvironment and vasculature, thereby facilitating chemotherapy drug absorption and reducing tumor metastasis [1].

Avastin®, initially approved by the U.S. Food and Drug Administration (FDA) in 2004 in combination with chemotherapy for the treatment of metastatic colorectal cancer (mCRC) as a first- or second-line approach, was one of the first targeted therapies and the first approved angiogenesis inhibitor. In 2005, the European Union (EU) granted Avastin® marketing authorization for the first-line treatment of patients with mCRC combining with chemotherapy. Since then, a variety of oncology indications have been approved worldwide, including but not limited to mCRC, non-squamous non-small cell lung cancer (NSCLC), metastatic renal cell carcinoma, glioblastoma multiforme (the U.S. only), epithelial ovarian cancer, fallopian tube cancer, cervical cancer, primary peritoneal cancer, and metastatic breast cancer (EU only) [2, 3]. In February 2010, the National Medical Products Administration (NMPA) authorized it for import registration with the indication of mCRC, followed by approval for the treatment of NSCLC in 2015. Lung cancer and colorectal cancer are two malignant tumors that affect many people worldwide. Lung cancer is expected to be the leading cause of death in China by 2022, followed by CRC, gastric cancer, liver cancer, and breast cancer [4].

Although bevacizumab has been approved for various tumor indications and its efficacy has been demonstrated, its high treatment cost limits its use by most patients with cancer [5, 6]. The introduction of biosimilars reduces the cost of medication and, more importantly, provides clinical benefits to patients while ensuring the safety and efficacy of treatment. As the patents on original foreign drugs expire, biosimilars’ research and development based on the authentic products’ quality, safety, and efficacy continue apace.

Biosimilars are therapeutic biologic products that are highly similar in product purity, safety, or potency to a licensed biologic (i.e., reference or originator) [7]. Shanghai Institute of Biological Products Co., Ltd. is developing a recombinant humanized anti-VEGF monoclonal antibody injection as a promising biosimilar to Avastin® (Roche Diagnostics GmbH, Germany). The biosimilar and Avastin® share the same primary amino acid sequence. The two products are very similar in expression host cells, main process steps, product quality, as well as stability. Preclinical pharmacology and toxicology studies have also demonstrated that the biosimilar and Avastin® are similar in terms of pharmacodynamics, pharmacokinetics (PK), and safety evaluation. The clinical development of the biosimilar was aided by the results of the analytical and preclinical in vivo studies. Following the guidelines set forth by the NMPA [8], a randomized, double-blind, single-dose, parallel-group phase I study was designed to demonstrate the bioequivalence between the recombinant humanized anti-VEGF monoclonal antibody injection and Avastin®.

## Methods

### Subjects

Eligible subjects were healthy men, aged 18-45 years, with a body mass index of 18–26 kg/m^2^ inclusive and body weight of 50–80 kg inclusive; agree to take effective contraceptive measures from signing the informed consent form until 6 months after the infusion of the study drug.

The main exclusion criteria were as follows: (1) abnormal clinical manifestations such as the nervous system, cardiovascular system, blood and lymphatic system, immune system, digestive system, respiratory system, metabolism, bone, and other systematic diseases; (2) hypersensitivity to recombinant anti-VEGF humanized monoclonal antibody injection, Avastin® and its excipients; (3) history of antibody therapy such as bevacizumab or VEGF-targeted drugs; (4) use of any biological product or been inoculated with a live attenuated vaccine within three months of the study drug infusion or use of any monoclonal antibody within nine months; (5) participation in any clinical trial within the previous three months before signing the informed consent form; (6) history of donating and/or receiving any blood or blood products, or massive blood loss (> 450 mL) in the last three months, or plan to donate blood during the study; (7) unhealed wound ulcers or fractures or history of major surgery within two months of randomization or plan to undergo significant surgery during the study or within two months after study completion; (8) positive alcohol breath test on the day of screening or admission or a history of alcohol abuse within three months prior to screening; (9) history of smoking > 5 cigarettes per day within three months prior to enrollment.

### Study design and ethics

According to the design of general bioequivalence study, due to the long half-life (18-20 days) and immunogenicity characteristics of bevacizumab [2, 3], this trial follows a randomized, double-blind, parallel-group design strategy [7–10]. The study was performed at Phase I Clinical Research Center of the Affiliated Hospital of Bengbu Medical College and conducted in accordance with the Declaration of Helsinki, Good Clinical Practice (GCP) and applicable laws and regulations of NMPA. This trial was registered in www.chinadrugtrials.org.cn, CTR20191923, and approved by the Clinical Medical Research Ethics Committee of the First Affiliated Hospital of Bengbu Medical College. Before undergoing study-specific procedures, all subjects signed informed consent form.

Previous studies indicated that the interindividual coefficient of variation (CV) of AUC_0–t_ after bevacizumab treatment was approximately 25% [11]. The equivalent standard is that the 90% confidence interval (CI) of the AUC_0–t_ geometric mean ratio (GMR) of the test drug and the reference drug was within the prespecified bioequivalence interval of 80.00%–125.00%, with a power set at 90%. The theoretical ratio of the GMR was 0.95, and the calculated sample size was 74 cases. A total of 88 healthy male subjects (44 subjects per arm) were planned to be recruited, assuming a 15% dropout and rejection rate.

All qualified study subjects were admitted to our institution’s Phase I Clinical Research Center on day 1 (prior to administration). They ate a standard meal in the evening and fasted for 10 h before the study. Water intake was restricted from 1 h before the infusion to 1 h after the infusion. According to the prescribing instructions [2, 3] for Avastin® and relevant reference [12], to prevent the occurrence of infusion-related reactions, it is recommended that small doses of antiallergic drugs such as chlorpheniramine and dexamethasone should be given properly prior to the infusion of bevacizumab. In current study, all subjects were given 4 mg of chlorpheniramine orally, followed by 5 mg of dexamethasone intravenously prior to receiving the study products [12]. In case of severe allergic reaction, the infusion of bevacizumab should be stopped immediately, and anti-allergic drugs should be given according to the doctor's prescriptions, and the changes of people’s vital signs should be monitored until the symptoms are completely relieved.

### Assessments

Subjects were screened following the protocol and then randomly assigned to either the test group or the reference group with a single dose of 3 mg/kg of each group administered intravenously for pharmacokinetic** (**PK) and immunogenicity (anti-drug antibody/neutralizing antibody (ADA/NAb) analysis.

Blood samples for PK analysis were collected at predose, immediately after the completion of the infusion (within 2 min), followed by 0.75, 2.5, 3.5, 9.5, 13.5, 24, 72, 168, 336, 504, 672, 840, 1008, 1344, and 1680 h after the infusion was completed. There were 17 sampling points in total, with each sample containing 2 ml. Serum samples were stored at − 80 °C, and the serum drug concentration was determined using a validated enzyme-linked immunosorbent assay.

Blood samples for ADA/NAb assessment were collected at predose, followed by 336, 672, 1008, and 1680 h after the infusion was completed. There were five blood collection points in total, with each sample containing 3 ml. Until the assay, samples were stored at -80 °C.

### Statistical analysis

#### Pharmacokinetics

The PK parameters assessed in this study include: (1) Primary endpoint: area under the concentration-time curve (AUC) from time zero to the last quantifiable concentration (AUC_0–t_); (2) Secondary endpoints: AUC from time zero to infinity (AUC_inf_), maximum serum concentration (C_max_), time to C_max_ (T_max_), apparent volume of distribution (Vd), terminal elimination rate constant (λ_z_), terminal half-life (t_1/2_), and drug clearance (CL). The PK parameters were estimated and analyzed using a non-compartmental model (WinNonlin, version 8.2). The main PK parameters were calculated to reflect the profiles of absorption, distribution, metabolism, and excretion of drugs in humans. Other statistical analyses were performed using Statistical Analysis System (version 9.4) software.

For evaluating equivalence in the primary PK parameters, the bioequivalence set (BES) and AUC_0–t_ were log-transformed and then subjected to a two-sided t-test to calculate the difference between the test group and the reference group as well as the 90% CI of the difference and finally the GMR of the PK parameters of the test group and reference group. The 90% CI of the ratio was calculated by taking the antilogarithm. Bioequivalence was established when the 90% CI of the GMR of AUC_0–t_ between the two groups was within 80.00%–125.00%. As necessary, we performed sensitivity analysis.

For the analysis of secondary PK parameters, modified bioequivalence set (mBES), C_max_, and AUC_inf_ were logarithmically transformed to perform a two-sided t-test. The difference in PK parameters between the test group and the reference group, and the 90% CI of the difference were calculated, followed by the GMR of the PK parameters of the test group and reference group, as well as the 90% CI of the ratio. The difference between T_max_ and t_1/2_ between the two groups was calculated using the Wilcoxon rank-sum test. The above PK parameter calculation results were only for reference and were not used to determine bioequivalence.

#### Safety

Adverse events (AEs), infusion-related reactions, physical examination, vital signs, 12-lead ECG, and immunogenicity were all evaluated for safety. The severity of the AEs was graded according to Common Terminology Criteria for AE (CTCAE). A treatment-emergent AE (TEAE) was defined as any event that occurred after the study drug was administered but was not present prior to administering the study drug.

## Results

### Baseline characteristics

**Fig. 1:**
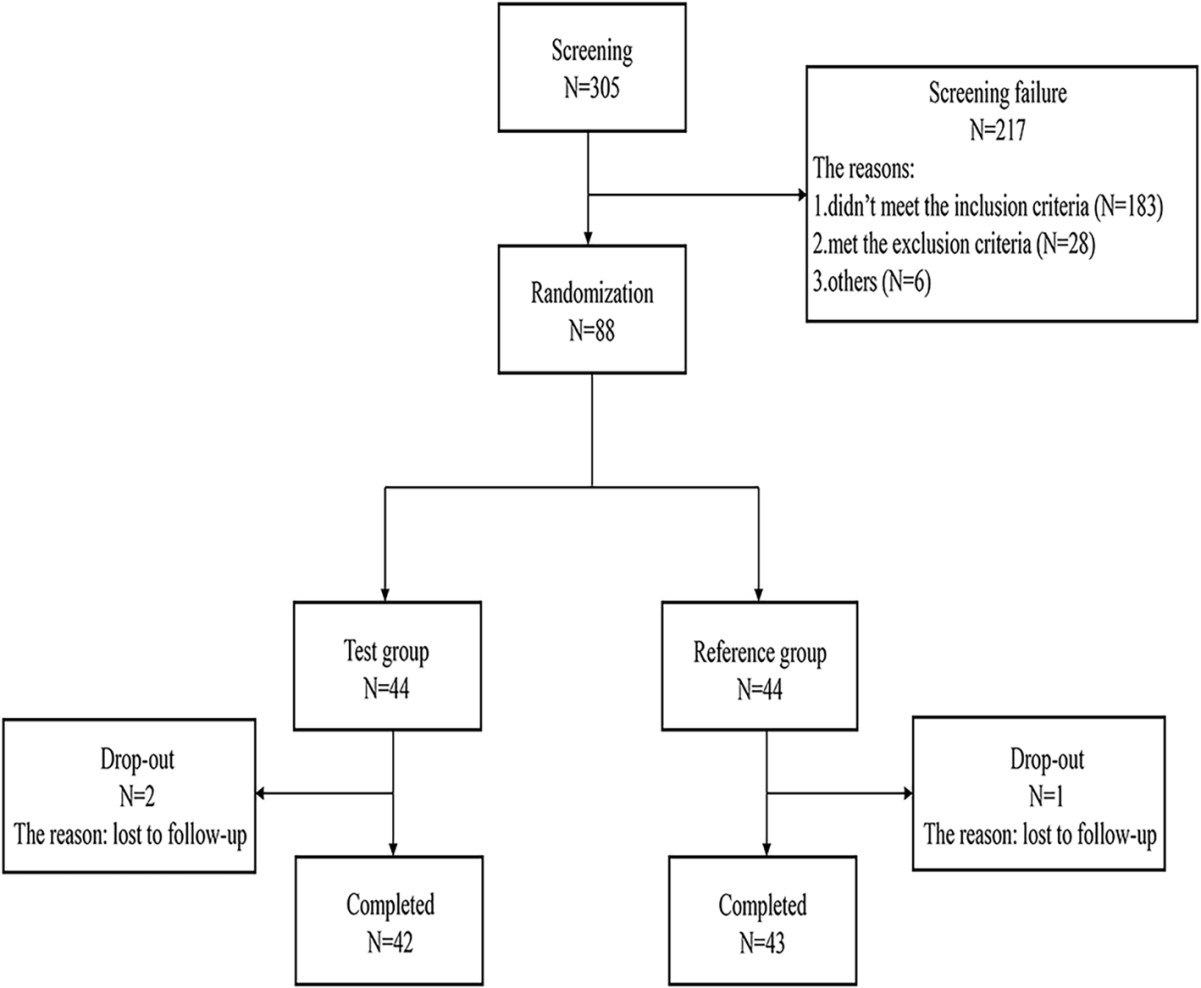
Study participants flow

Of the 305 subjects screened, 217 failed to pass, and 88 (44 per treatment arm) were enrolled. Three subjects dropped out due to “lost to follow-up,” but the remaining 85 completed the trial (Fig. 1). Subject 014, in the test group, dropped out on day 36, so the blood drug concentration data were missing from this visit till the end-of-study (EOS) visit (i.e., hours 840, 1008, 1344, and 1680 after the infusion was completed). Subject 041, in the reference group, dropped out on day 15, so the blood drug concentration data were missing from this visit till the EOS visit (i.e., hours 336, 504, 672, 840, 1008, 1344, and 1680 after the infusion was completed). Subject 014 and 041 lost the concentration data of more than three consecutive blood sampling points, causing the PK parameters AUC_0-t_, AUC_inf_, AUC__%Extra_ obs_, t_1/2_, λ_Z_, CL and Vd to become invalid, while C_max_ and T_max_ are valid. Subject 014 and 041 can be included in the pharmacokinetic parameter set (PKPS), while excluded from the BES. Subject 072, in the test group, dropped out on day 57, so the blood drug concentration data were missing from this visit till the EOS visit (i.e., hours 1344, and 1680 after the infusion was completed). Subject 072 can be included in both the PKPS and BES. Among the treatment groups, the baseline characteristics of subjects were comparable (Table 1).

**Table 1:** Baseline characteristics

Items	Test group *N* = 44	Reference group *N* = 44	Total *N* = 88
Gender, n (%)			
Male	44 (100.0)	44 (100.0)	88 (100.0)
Age (years)	28.7 (5.7)	28.6 (5.9)	28.7 (5.8)
Race, n (%)			
Han Chinese	43 (97.7)	43 (97.7)	86 (97.7)
Non-Han Chinese	1 (2.3)	1 (2.3)	2 (2.3)
Height (cm)	170.30 (5.44)	170.19 (5.68)	170.24 (5.53)
Body weight (kg)	62.87 (6.41)	61.63 (5.99)	62.25 (6.20)
BMI (kg/m^2^)	21.68 (2.00)	21.28 (1.86)	21.48 (1.93)

BMI (kg/m^2^) = weight (kg)/(height [m])^2^

*BMI* Body mass index, Data are Mean (SD), *SD* Standard deviation

### Pharmacokinetics

**Fig. 2:**
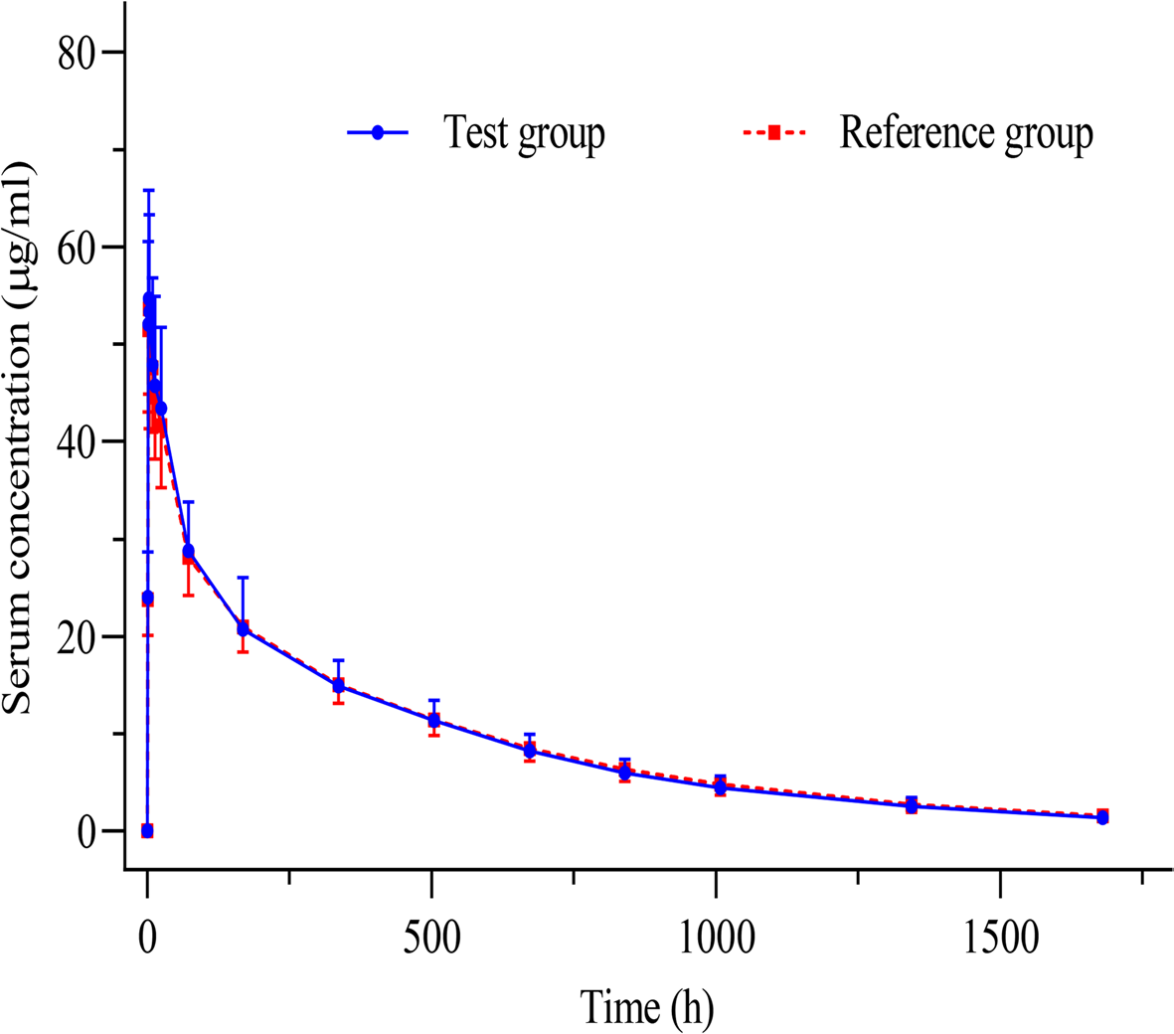
Mean (SD) serum concentration-time profiles

The mean serum concentration–time profiles for the two groups are shown in Fig. 2. The figure shows that the mean value of serum drug concentration in the test group and reference group was similar at each time point, with no significant differences. Table 2 summarizes the specific PK parameters of the test group and the reference group. The median T_max_ of each group was 2.50 h; the average C_max_, AUC_0–t_, and AUC_inf_ of the two groups were 56.65 μg/mL and 55.63 μg/mL, 15,914.66 μg/mL·h and 16,202.44 μg/mL·h, and 16,803.51 μg/mL·h and 17,144.05 μg/mL·h, respectively.

**Table 2:** Summary of pharmacokinetic parameters

Parameters	Mean ± SD (%CV)			
	Test group		Reference group	
T_max_^a^ (h)	*N* = 44	2.50 (1.50,13.50) (71.38)	*N* = 44	2.50 (1.46,9.51) (45.83)
C_max_ (μg/mL)	*N* = 44	56.65 ± 10.20 (18.00)	*N* = 44	55.63 ± 8.53 (15.33)
AUC_0-t_ (μg/mL•h)	*N* = 43	15,914.66 ± 2874.42 (18.06)	*N* = 43	16,202.44 ± 2025.34 (12.50)
AUC_inf_ (μg/mL•h)	*N* = 43	16,803.51 ± 3243.53 (19.30)	*N* = 43	17,144.05 ± 2433.10 (14.19)
λ_z_ (1/h)	*N* = 43	0.0019 ± 0.0004 (19.57)	*N* = 43	0.0018 ± 0.0003 (16.86)
t_1/2_ (h)	*N* = 43	389.42 ± 83.86 (21.53)	*N* = 43	402.79 ± 72.17 (17.92)
CL (L/h)	*N* = 43	0.0117 ± 0.0025 (21.33)	*N* = 43	0.0111 ± 0.0016 (14.67)
Vd (L)	*N* = 43	6.38 ± 1.12 (17.54)	*N* = 43	6.34 ± 1.02 (16.02)

*Note*: Subject 014 and 041 lost the concentration data of more than three consecutive blood sampling points, causing the PK parameters AUC_0-t_, AUC_inf_, AUC__%Extra_ obs_, t_1/2_, λ_Z_, CL and Vd to become invalid, while C_max_ and T_max_ are valid. Subject 014 and 041 can be included in the pharmacokinetic parameter set (PKPS), while excluded from the BES

For subject 017, in the reference group, the infusion discontinued at 77 min and 27 s (about 1.291 h) after dosing, and blood collection was performed immediately (unplanned blood sampling), with the blood drug concentration of 38.8 μg/mL. The PK parameters presented in this table were calculated by including the concentration data of the unplanned blood sampling point. In addition, the PK parameters did not change when eliminating the concentration data of the unplanned blood sampling point, except the AUC_0-t_ and AUC_inf_ values

*AUC*_*0-t*_ Area under the serum concentration–time curve (AUC) from time zero to last quantifiable concentration, *AUC*_*inf*_ AUC from time zero to infinity, *C*_*max*_ Maximum serum concentration, *CV* Coefficient of variation, *SD* Standard deviation, *T*_*max*_ Time to C_max_, *T*_*1/2*_ Terminal half-life, *Vd* Apparent volume of distribution, *λ*_*z*_ terminal elimination rate constant

^a^T_max_ is expressed by median (minimum, maximum)

### The main analysis results of the equivalence evaluation

The GMR of AUC_0–t_, C_max_, and AUC_inf_ between the test group and the reference group had 90% CI of 91.71%–103.18%, 95.72%–107.49%, and 91.03%–103.43%, respectively. All of these values fell within the equivalence interval of 80.00%–125.00%. Therefore, we established the bioequivalence between the two arms (Table 3). The median t_1/2_ of test group and reference group were 378.55 h and 388.72 h, respectively. The median T_max_ of both groups were 2.50 h. The Wilcoxon rank-sum test results showed no significant difference between the two groups.

**Table 3:** Equivalence of C_max_, AUC_0-t_, and AUC_inf_ (BES)

Parameters	Geometric mean and ratio							
	Test group (T)		Reference group (R)		T/R (%)	Individual variation among subjects CV (%)	90% CI %^a^	Power (%)
C_max_ (μg/mL)	*N* = 44	55.80	*N* = 44	55.01	101.43	16.48	95.72 ~ 107.49	> 99.99
AUC_0-t_ (μg/mL•h)	*N* = 43	15,639.80	*N* = 43	16,077.68	97.28	16.52	91.71 ~ 103.18	> 99.99
AUC_inf_ (μg/mL•h)	*N* = 43	16,472.86	*N* = 43	16,976.75	97.03	17.95	91.03 ~ 103.43	> 99.96

*Note*: Subject 014 and 041 lost the concentration data of more than three consecutive blood sampling points, causing the PK parameters AUC_0-t_, AUC_inf_, AUC__%Extra_ obs_, t_1/2_, λ_Z_, CL and Vd to become invalid, while C_max_ and T_max_ are valid. Subject 014 and 041 can be included in the pharmacokinetic parameter set (PKPS), while excluded from the BES

AUC_0-t_ and AUC_inf_ for subject 017 (the reference group) were calculated by using the concentration data at the unplanned blood sampling point

*AUC*_*0-t*_ Area under the serum concentration–time curve (AUC) from time zero to last quantifiable concentration, *AUC*_*inf*_ AUC from time zero to infinity, *CI* Confidence interval, *C*_*max*_ Maximum serum concentration, *CV* Coefficient of variation

^a^is the 90% CI for the geometric mean ratio of T/R

### Results of sensitivity analysis for equivalence evaluation

Based on the main analysis, the invalid PK parameters of subjects 014 and 041 were re-incorporated. Subject 070, in the reference group, took concomitant medication once during the trial, but there was no evidence that the concomitant medication affected the drug pharmacokinetics, so the PK parameters were valid and can be included in both PKPS and BES. When performing the sensitivity analysis, the AUC-related parameters of subject 070 (including AUC_0–t_, AUC_inf_, and t_1/2_) were eliminated. The PK parameters of subject 017 were replaced by the PK parameters calculated by removing the concentration data at the unplanned blood sampling point. Then we performed the sensitivity analysis of the equivalence evaluation. The GMR of AUC_0–t_ and AUC_inf_ between the test group and the reference group were 92.72%–108.68% and 92.69%–106.97%, respectively, falling within the equivalence interval 80.00%–125.00% and establishing bioequivalence (Table 4). The median t_1/2_ of test group and reference group were 377.63 h and 384.66 h, respectively. The Wilcoxon rank-sum test results showed no significant differences between the two groups.

**Table 4:** Sensitivity analysis of AUC_0-t_ and AUC_inf_ equivalence evaluation (mBES)

Parameters	Geometric mean and ratio							
	Test group (T)		Reference group (R)		T/R (%)	Individual variation among subjects CV (%)	90% CI %^a^	Power (%)
AUC_0-t_ (μg/mL•h)	*N* = 44	15,537.00	*N* = 43	15,477.67	100.38	22.54	92.72 ~ 108.68	99.71
AUC_inf_ (μg/mL•h)	*N* = 44	16,447.82	*N* = 43	16,518.34	99.57	20.29	92.69 ~ 106.97	99.95

*Note*: AUC_0-t_ and AUC_inf_ of subject 017 (the reference group) were calculated by removing the concentration data at the unplanned blood sampling point; AUC_0-t_ and AUC_inf_ of subjects 014 (the test group) and 041 (the reference group) not included in BES were re-incorporated, and AUC_0-t_ and AUC_inf_ of subject 070 (the reference group) were eliminated

*AUC*_*0-t*_ Area under the serum concentration–time curve (AUC) from time zero to last quantifiable concentration, *AUC*_*inf*_ AUC from time zero to infinity, *CI* Confidence interval

^a^is the 90% CI for the geometric mean ratio of T/R

### Safety

All 88 subjects in this trial entered the Safety Analysis Set (SS) and completed the administration as per the protocol. A total of 81 subjects experienced 306 AEs, with one subject experiencing “skin redness” prior to receiving the test drug; the remaining 81 subjects (305 cases) were treatment-emergent AEs (TEAEs), with an incidence rate of 92.05%; 40 subjects (152 cases) in the test group had AEs, with an incidence rate of 90.91%; and 41 subjects (153 cases) in the reference group had AEs, with an incidence rate of 93.18%. Adverse reactions (ARs) occurred in 80 subjects (253 cases), with an incidence rate of 90.91%; 40 cases (124 subjects) in the test group had ARs, with an incidence rate of 90.91%; and 40 subjects (129 cases) in the reference group had ARs, with an incidence rate of 90.91%; and one case (1.14%) had an infusion reaction. The two groups had similar rates of AEs and ARs. No SAE, suspected unexpected serious ARs, AEs of particular concern, or AEs that resulted in the study’s termination were noted.

AEs that occurred during the trial were summarized using system organ classification and preferred term: at least one AE occurred in 40 subjects in the test group, with an incidence rate of 90.91%; 41 subjects in the reference group experienced at least one AE, with an incidence rate of 93.18%. The AEs that occurred in the two groups were “various examinations” (the incidence of AEs in the test group and the reference group was 84.09% and 75.00%, respectively), “metabolic and nutritional diseases” (the incidence of AEs in the two groups was 45.45% and 61.36%, respectively), and “respiratory system, thoracic, and mediastinal diseases” (the incidence of AEs in the two groups was 4.55% and 18.18%, respectively) (Table 5).

**Table 5:** Summary of adverse events

System organ class	Test group (*N* = 44)		Reference group (*N* = 44)		Total (*N* = 88)	
Preferred term	Cases (incidence %)	Frequency	Cases (incidence %)	Frequency	Cases (incidence %)	Frequency
Total	40 (90.91)	152	41 (93.18)	153	81 (92.05)	305
Various inspections	37 (84.09)	105	33 (75.00)	81	70 (79.55)	186
Elevated white blood cell count	25 (56.82)	29	19 (43.18)	19	44 (50.00)	48
Elevated neutrophil count	15 (34.09)	16	12 (27.27)	12	27 (30.68)	28
Positive occult blood	8 (18.18)	8	6 (13.64)	6	14 (15.91)	14
positive urine white blood cells	4 (9.09)	5	7 (15.91)	8	11 (12.50)	13
positive urine red blood cell positive	4 (9.09)	4	4 (9.09)	8	8 (9.09)	12
Elevated blood bilirubin	6 (13.64)	8	1 (2.27)	1	7 (7.95)	9
Metabolic and nutritional diseases	20 (45.45)	36	27 (61.36)	42	47 (53.41)	78
Hypertriglyceridemia	13 (29.55)	15	14 (31.82)	16	27 (30.68)	31
Hyperglycemia	11 (25.00)	12	9 (20.45)	9	20 (22.73)	21
Hypokalemia	2 (4.55)	2	7 (15.91)	12	9 (10.23)	14
hyperuricemia	6 (13.64)	6	2 (4.55)	2	8 (9.09)	8
Respiratory, thoracic and mediastinal diseases	2 (4.55)	3	8 (18.18)	9	10 (11.36)	12
Runny nose	2 (4.55)	3	3 (6.82)	3	5 (5.68)	6
Infectious and infectious diseases	2 (4.55)	2	5 (11.36)	5	7 (7.95)	7
Upper respiratory tract infection	2 (4.55)	2	4 (9.09)	4	6 (6.82)	6
Gastrointestinal diseases	0	0	6 (13.64)	8	6 (6.82)	8
Skin and subcutaneous diseases	2 (4.55)	2	2 (4.55)	4	4 (4.55)	6
Kidney and urinary diseases	2 (4.55)	2	1 (2.27)	1	3 (3.41)	3
Heart organ disease	2 (4.55)	2	1 (2.27)	1	3 (3.41)	3
Various neurological diseases	0	0	2 (4.55)	2	2 (2.27)	2

The vital signs of all subjects were primarily stable throughout the trial; 8 had clinically significant physical examination abnormalities, 77 had clinically significant laboratory abnormalities, and 4 had clinically significant abnormal 12-lead ECG. These abnormalities were mild, and returned to normal without intervention. The chest X-ray, abdominal ultrasonography, and urinary system ultrasonography were normal or abnormal with no clinical implications.

### Immunogenicity

The immunogenicity analysis set included a total of 87 subjects. Among the 44 subjects in the test drug group, only 1 subject (random number 039) tested positive for ADA on day 71. The test results of this subject at other time points and the other 43 subjects were tested negative. Two of the 43 subjects in the reference group (random numbers 005 and 051) tested positive for ADA (005 on day 29 and 051 on day 43). The test results of these 2 subjects at the remaining time points and the test results of the other 41 subjects at all time points were negative. In the three cases above, the titers corresponding to positive ADA test results were all 1:50, and the corresponding NAb results were negative. In both treatment groups, the incidence of ADA was low, and subjects with positive ADA had little effect on overall PK parameters.

## Discussion

In this study, the recombinant humanized anti-VEGF monoclonal antibody injection and Avastin® had similar PK, clinical safety, and immunogenicity characteristics after a single intravenous infusion.

The test drug and the reference drug had similar mean serum concentration-time profiles. The 90% CIs of the GMR of AUC_0–t_, C_max_, and AUC_inf_ in the two groups were 91.71%–103.18%, 95.72%–107.49%, and 91.03%–103.43%, respectively, all falling within the equivalence margin of 80%–125.00%. These results were in accordance with previous studies on bevacizumab [13–24].

The PK of bevacizumab was linear between 1 and 10 mg/kg [2, 3, 11]. According to NMPA’s biosimilar guidelines [8], choosing a lower dosage to reduce drug exposure to healthy subjects is recommended from an ethical standpoint. Taking into account the lower limit of quantification of the applied method for drug concentration measurement, as well as other factors, this study ultimately chose a subtherapeutic dose of 3 mg/kg, which is lower than the clinically used dose, reducing the risk of AEs in healthy volunteers, while still obtaining useful PK data. Furthermore, this dose was consistent with the dose used in other similar phase I clinical studies conducted worldwide [13–24].

According to associated guidelines [7–9], healthy volunteers are an ideal homogeneous population for evaluating the PK difference between a biologic drug and a reference drug. Potential confounding factors, such as disease stages and prognosis, disease-specific complications, or concomitant medications, may increase variability in PK parameters in patients with cancer.

The most common AEs reported in previous studies on bevacizumab biosimilars performed in healthy subjects were headache, nasopharyngitis, pharyngitis, upper respiratory tract infection, hypertriglyceridemia, hypokalemia, nausea, and diarrhea [13–24]. Hypertension, fatigue or asthenia, diarrhoea and abdominal pain were the most frequently observed adverse reactions receiving bevacizumab in patients with cancer [3]. In this study, increased white blood cell count, hypertriglyceridemia, increased neutrophil count, hyperglycemia, positive occult blood, hypokalemia, and positive urine white blood cells were the most commonly reported AEs, occurring in > 10% of participants. The different AEs in our study could be due to differences in the subjects’ race, age, BMI, as well as sample size. No SAE occurred, and no subject withdrew due to an AE. The study drugs were well tolerated, with no new or unexpected safety concerns.

Throughout the study, the incidence of ADAs was low and comparable in both groups, with only 1 of 44 subjects in the test group and 2 of 43 subjects in the reference group testing positive for ADAs. The results of the NAb tests were all negative. The lower incidence of immunogenicity observed in this study was in line with other studies on bevacizumab biosimilars and product characteristics [2, 3, 11, 13–24].

Overall, the laboratory results and other safety measures were unremarkable, and we identified no safety concerns. There were no clinically significant differences with respect to laboratory tests, vital signs, and ECGs between the treatment groups. Because bevacizumab was administrated at a low subtherapeutic dose in healthy subjects, the study’s safety results are only helpful for comparison.

One of the study’s limitations is that the study population is radically and ethnically homogenous. Only Chinese male subjects were selected, with Han nationality accounting for 97.73%. Another limitation is that the subjects only received a single injection of the recombinant humanized anti-VEGF monoclonal antibody injection or Avastin®. However, the PK data from this trial’s single administration will provide a foundation for further studies. A phase III study with multiple doses in larger patient population is required to further develop recombinant humanized anti-VEGF monoclonal antibody injection. Furthermore, previous studies have shown that gender influenced test results, and bevacizumab increased the risk of ovarian failure in women and might impair female fertility [2, 3]. Therefore, only healthy male subjects were selected in this study, which was in line with other studies on bevacizumab biosimilars [11, 13–24]. In addition, in this study, only Avastin® sourced from Europe was selected for comparison, while both U.S. and Europe-sourced Avastin® were used in other bevacizumab biosimilar studies [13, 14, 18, 21]. This is because Avastin® registered in China was manufactured in Europe when conducting this current study. In the future, we may conduct a study to compare the recombinant humanized anti-VEGF monoclonal antibody injection with Avastin® from other sources, such as the United States.

## Conclusions

The current study demonstrated that the recombinant humanized anti-VEGF monoclonal antibody injection was bioequivalent to Avastin®. The safety and immunogenicity were similar between the two groups. Subsequent studies should investigate recombinant humanized anti-VEGF monoclonal antibody injection in patients setting.

## Supplementary Information

**Additional file 1.**

## Data Availability

The data that support the findings of this study are available from the corresponding author upon reasonable request.

## Abbreviations

ADA: Anti-drug antibody
AE: Adverse event
ANOVA: Analysis of variance
ARs: Adverse reactions
AUC_0-t_: Area under the concentration-time curve from time zero to the last measurable concentration
AUC_inf_: Area under the concentration-time curve from time zero to infinity
BES: Bioequivalence set
BMI: Body mass index
C_max_: Maximum concentration
CI: Confidence interval
CL: Clearance
CTCAE: Common Terminology Criteria for AE
CV: Coefficient of variation
ECGs: Electrocardiograms
GCP: Good Clinical Practice
GMR: Geometric mean ratio
mBES: Modified bioequivalence set
NAbs: Neutralizing antibodies
PK: Pharmacokinetics
PKPS: Pharmacokinetic parameter set
RP: Reference product
SAE: Serious adverse event
SD: Standard deviation
SS: Safety Analysis Set
TEAE: Treatment-emergent adverse event
T_1/2_: Elimination half-life
T_max_: Time to peak drug concentration
Vd: Apparent volume of distribution
λz: Terminal elimination rate constant
