# Supplementary material for: A phase I study comparing the biosimilarity of the pharmacokinetics and safety of recombinant humanized anti-vascular endothelial growth factor monoclonal antibody injection with Avastin® in healthy Chinese male subjects
Source: BMC Pharmacol Toxicol. 2023 May 27;24:36. doi: 10.1186/s40360-023-00673-y (PMC10223897; doi:10.1186/s40360-023-00673-y)
Supplement: Supplementary file 1 — Additional file 1. [file 40360_2023_673_MOESM1_ESM.docx]

**2.2 Subjects**

**2.2.1 Inclusion criteria**

1. Subjects who agreed to participate in this trial sign the informed consent form.
2. Healthy male participants aged 18–45 years, with a body mass index (BMI) of 18–26 kg/m^2^ (including 18 kg/m^2^ and 26 kg/m^2^) and body weight of 50–80 kg (including 50 kg and 80 kg).
3. Vital signs, physical examination, laboratory tests (including but not limited to blood routine, blood biochemistry, coagulation function, urine routine, and fecal occult blood test), 12-lead electrocardiogram (ECG), chest X-ray, abdominal ultrasound, and urinary ultrasound examinations are normal. If not clinically significant, such abnormal tests are judged by the investigators.
4. Acceptance to use effective contraception (including but not limited to abstinence, physical contraception, or hormonal contraception) from the time the informed consent form is signed until six months after the study drug is infused (hormonal contraception is not allowed).
5. Willingness to complete all follow-up visits.

**2.2.2 Exclusion criteria**

1. Abnormal clinical manifestations such as the nervous system, cardiovascular system, blood and lymphatic system, immune system, digestive system, respiratory system, metabolism, bone, and other systematic diseases.
2. A history of gastrointestinal perforation or a gastrointestinal fistula disease.
3. Allergic to recombinant anti-VEGF humanized monoclonal antibody injection, Avastin^®^ and its excipients.
4. Known history of autoimmune disease, allergic disease, or food or drug allergy.
5. Clinically significant proteinuria (routine urinalysis, urine protein 2+ and above) determined by the investigator or a history of proteinuria.
6. History of hereditary bleeding or coagulation disorder, nontraumatic bleeding (bleeding that requires treatment), history of thromboembolism, and any disorder that could cause bleeding (including coagulation disorder, thrombocytopenia (platelet count <125 x 10^9^/L), and coagulation international normalized ratio >1.5).
7. Vigorous exercise for 96 h prior to the study drug infusion or engagement in vigorous physical activity throughout the trial, including contact or impact sports.
8. History of tumors in the family or malignant tumors in self.
9. Abnormal ECG or corrected QTc interval (QTc was calculated using Fridericia correction formula QTc = QT/(RR^0.33)> 450 ms), which was clinically significant as judged by the investigator.
10. Previous history of hypertension or abnormal blood pressure (systolic blood pressure ≤90 mmHg or ≥140 mmHg, and/or diastolic blood pressure ≤60 mmHg or ≥90 mmHg) at study screening and admission (1 day before dosing), as well as an abnormal pulse at <60 bpm or >100 bpm.
11. Acute or chronic infectious diseases that were clinically significant at screening and admission or tested positive for hepatitis C antibody, human immunodeficiency virus Ab, hepatitis B surface antigen, or syphilis.
12. History of antibody therapy such as bevacizumab or VEGF-targeted drugs.
13. Use of any biological product or been inoculated with a live attenuated vaccine within three months of the study drug infusion or use of any monoclonal antibody within nine months.
14. Treatment with prescription or non-prescription drugs, Chinese herbal medicines, or health care products within 28 days prior to randomization.
15. Participation in any clinical trial within the previous three months before signing the informed consent form.
16. History of donating and or receiving any blood or blood products, or massive blood loss (>450 mL) in the last three months, or plan to donate blood during the study.
17. Unhealed wound ulcers or fractures or history of major surgery within two months of randomization or plan to undergo significant surgery during the study or within two months after study completion.
18. Plan to receive treatment or dental surgery during the study period.
19. Positive alcohol breath test on the day of screening or admission or a history of alcohol abuse within three months prior to screening (with alcohol consumption exceeding 14 units per week [1 unit was equal to approximately 360 mL of beer or 45 mL of 40% alcohol or 150 mL of wine]).
20. History of drug use or drug abuse, or positive drug screening results.
21. History of smoking >5 cigarettes per day three months prior to enrollment.
22. Any other conditions considered inappropriate by the investigator.
